# Dried-Blood Spots: A Cost-Effective Field Method for the Detection of Chikungunya Virus Circulation in Remote Areas

**DOI:** 10.1371/journal.pntd.0002339

**Published:** 2013-07-25

**Authors:** Soa Fy Andriamandimby, Jean-Michel Heraud, Laurence Randrianasolo, Jean Théophile Rafisandratantsoa, Seta Andriamamonjy, Vincent Richard

**Affiliations:** 1 Virology Unit, Institut Pasteur de Madagascar, Antananarivo, Madagascar; 2 Epidemiology Unit, Institut Pasteur de Madagascar, Antananarivo, Madagascar; Tropical Medicine Institute Pedro Kourí (IPK), Cuba

## Abstract

**Background:**

In 2005, there were outbreaks of febrile polyarthritis due to Chikungunya virus (CHIKV) in the Comoros Islands. CHIKV then spread to other islands in the Indian Ocean: La Réunion, Mauritius, Seychelles and Madagascar. These outbreaks revealed the lack of surveillance and preparedness of Madagascar and other countries. Thus, it was decided in 2007 to establish a syndrome-based surveillance network to monitor dengue-like illness.

**Objective:**

This study aims to evaluate the use of capillary blood samples blotted on filter papers for molecular diagnosis of CHIKV infection. Venous blood samples can be difficult to obtain and the shipment of serum in appropriate temperature conditions is too costly for most developing countries.

**Methodology and principal findings:**

Venous blood and dried-blood blotted on filter paper (DBFP) were collected during the last CHIKV outbreak in Madagascar (2010) and as part of our routine surveillance of dengue-like illness. All samples were tested by real-time RT-PCR and results with serum and DBFP samples were compared for each patient. The sensitivity and specificity of tests performed with DBFP, relative to those with venous samples (defined as 100%) were 93.1% (95% CI:[84.7–97.7]) and 94.4% (95% CI:[88.3–97.7]), respectively. The Kappa coefficient 0.87 (95% CI:[0.80–0.94]) was excellent.

**Conclusion:**

This study shows that DBFP specimens can be used as a cost-effective alternative sampling method for the surveillance and monitoring of CHIKV circulation and emergence in developing countries, and probably also for other arboviruses. The loss of sensitivity is insignificant and involved a very small number of patients, all with low viral loads. Whether viruses can be isolated from dried blood spots remains to be determined.

## Author Summary

Chikungunya is a mosquito-transmitted viral disease. No treatment is currently available. The only way to prevent infection is to avoid mosquito bites. Surveillance of circulation by early diagnosis is useful to prevent or limit outbreak. CHIKV, like all RNA viruses, is heat-labile. Consequently, confirmatory diagnosis classically requires blood samples that are transported in appropriate conditions (i.e. at 4°C within 48 hours, in liquid nitrogen, or frozen at −80°C and transported on dry ice) to prevent false negative results. This is not always possible in field conditions in low income countries. Dried blood spots are already used to diagnose parasitic, bacterial and viral infection. We compared venous sample to dried blood sample to make diagnosis of Chikungunya infection. We demonstrate the usefulness of this sampling method for the molecular diagnosis of Chikungunya infection. In particular, dried blood spots were very nearly as suitable as frozen serum specimens for the diagnosis of recent infection by CHIKV.

## Introduction

Chikungunya virus (CHIKV) is an arthropod-borne alphavirus transmitted by mosquitoes of the *Aedes* genus. Chikungunya has spread widely through Africa [1], [2], [3], South-East Asia, the Indian subcontinent [4], [5], [6], and most recently Southern Europe [7], [8]. There was a large outbreak in La Réunion in 2005–2006 with an estimated 266,000 individuals infected [9]. Populations on other islands in the Indian Ocean (Mauritius, Madagascar, Mayotte, the Seychelles and islands of the Union of the Comoros) were also affected by this virus [3], [10], [11]. The Northern and Eastern parts of Madagascar experienced their first documented Chikungunya outbreak in 2006, concomitantly with the circulation of dengue virus serotype 1 [11]. Since then, CHIKV has become endemic and now circulates continually in the eastern part of the island. In February 2010, the primary health care centre of Mananjary (southeast of Madagascar) reported an outbreak of dengue-like illness (fever, arthralgia, myalgia, rash, and headache). Laboratory tests confirmed CHIKV infection in more than 90% of the patients sampled. Due to logistic constraints and lack of resources, it was decided to monitor the outbreak using an alternative support for blood specimens. Arboviruses are heat-labile RNA viruses, and diagnosis of these virus traditionally requires a blood sample stored at 4°C for only a short time, and quickly transported to the laboratory at a temperature not exceeding 4°C [12]. Dried-blood blotted on filter papers (DBFP) are a possible alternative, cost-effective and technically appropriate for low-incomes countries. Several studies have demonstrated that DBFP are suitable for serological and molecular diagnosis of bacterial, viral, and parasitic diseases [13], [14], [15], [16], . The diagnosis of dengue infection using DBFP has already been described [21], [22], this support has not been evaluated for the diagnosis of Chikungunya, other than during a serological study [23]. During a CHIKV outbreak on the East coast of Madagascar, we evaluate the value of DBFP for the molecular diagnosis of CHIKV infection and its usefulness for monitoring a CHIKV outbreak in a low-resources country.

## Materials and Methods

### Ethical considerations

Specimens and data were collected within the activities of the national public health sentinel surveillance systems and therefore this was considered to be non-research activity. Fever surveillance, including arbovirus protocols, was approved by the respective ministries of health and the National Ethics Committee of Madagascar (FWA00016900). Before taking each specimen, physicians explained the purpose of the surveillance system. Patients were then free to refuse to participate. Oral consent was documented in the patient form. This research did not cause any additional trauma and all injuries suffered by individuals were associated with routine care. The specimens used in this study were collected as part of the routine care, and this study was designed retrospectively. As specimens were anonymous, written consent could not be obtained. The National Ethics Committee approved the use of oral consent.

### Human surveillance system

In 1996, in accordance with World Health Organisation resolution AFR/RC43/R7, the Integrated Diseases Surveillance and Response system was implemented by the ‘Direction des Urgences et de la Lutte contre les Maladies Négligées’ (DULMN) of the Malagasy Ministry of Public Health. Chikungunya fevers are a notifiable disease. To detect such event, a sentinel surveillance network for fever syndrome was established in 2007 and now includes 34 sentinel sites in 32 health districts of Madagascar [24]. All patients presenting at one clinic with fever were tested for malaria using a rapid diagnostic test (RDT); the general practitioner filled case report forms for all febrile patients. In some clinics involved in virological surveillance, patients that fulfilled the case definition for dengue-like illness with onset of fever 5 or fewer days earlier were sampled. Specimens were stored in a liquid nitrogen tank and shipped weekly to the National Reference Laboratory for Arboviruses (NRLA) at the Institut Pasteur from Madagascar.

### Specimen collection

Specimens used in this study were collected during two different periods. The first period (from February to March 2010) was during an outbreak of Chikungunya in two districts on the Southeast coast of Madagascar (Mananjary and Farafangana). The second period was the post-outbreak period (from July to September 2010). All patients, visiting sentinel centres, that fulfilled the case definition for dengue-like illness with onset within the previous 5 days were included in the study and both venous blood and capillary blood spotted onto a clean Whatman 3MM filter paper (Sigma-Aldrich, St. Louis, MO, USA) were collected. Dengue-like illness was defined as a presence of fever ≥38°C and two or more of the following: retro-orbital or ocular pain, headache, rash, myalgia, arthralgia, leukopenia, and haemorrhagic manifestations [25]. Cases and controls were defined retrospectively as follows: sera from patients included in our study and who fulfilled the dengue-like syndrome case definition were tested for Chikungunya. Cases were then defined as patients with Chikungunya infection confirmed by real-time RT-PCR or Indirect Immunofluorescence assay after viral isolation on Vero E6 or AP61 cells. Controls were defined as patients included in the study but tested negative for Chikungunya. All sera were kept in a liquid nitrogen tank before shipping to the NLRA then at −80°C from arrival until analysis. DBFP were kept at room temperature (25°C) until analysis as previously described [26].

### Detection of Chikungunya virus by real time RT-PCR from sera and DBFP positive controls

CHIKV RNA was obtained after propagation of CHIKV in Vero E6 cells for 5 days. Cell supernatants, enriched with CHIKV (CHIKV-SP), were then collected and a volume of 100 µL was tested to confirm the presence of CHIKV (see below).

### RNA extraction

RNA was extracted from 100 µL aliquots of serum or CHIKV-SP, using Trizol LS (Invitrogen Life Technologies, Paisley, Refrewshire, United Kingdom), according to the manufacturer's recommendations. The RNA was precipitated with 400 µL of isopropanol (SIGMA), air-dried and suspended in 50 µL of RNase-free water.

For DBFP, a 6 mm diameter disk containing the dried blood spot was cut using a paper puncher and placed in a 1.8 mL tube as previously described [27]. To avoid contamination and false positive results, puncher was soaked in NaOH (O.1N) and rinsed with RNase free water between each DBFP. Viral RNA was then extracted using Trizol reagent (Invitrogen Life Technologies, Paisley, Refrewshire, United Kingdom), according to the manufacturer's recommendations. RNA was precipitated with 500 µL of isopropanol. To validate each series of extraction, 15 µL of CHIKV-SP was spotted onto Whatman 3 MM filter Papers and dried. Six mm diameter disks were cut and put in a sealed plastic bag to prevent moistening and stored at +4°C until use as controls [26]. DBFP from healthy individuals previously tested negative for CHIKV were used as negative controls.

### Viral RNA amplification

CHIKV RNA was detected by one-step real-time RT-PCR assays in a Rotorgene 6000 apparatus (Corbett life science). Oligonucleotide primers were used with dual-labelled hydrolysis (Taqman) probes adapted from Laurent P et al, that target the E1 region (GenBank AF369024) (Table 1) [28]. The Ag-Path One Step RT-PCR kit (P/N: 4387391, Ambion, Foster City, CA, USA) was used for amplification. The reaction mix, in a final volume of 25 µl, consisted of 2.5 µL of RNA extract, primers at a final concentration of 0.5 µM, and the CHIK probe at a final concentration of 0.3 µM. The RT-PCR conditions were as follows: a 40 min reverse-transcription step at 50°C followed by denaturation for 10 min at 95°C and 45 cycles of denaturation at 95°C for 10 s and annealing/extension at 56°C for 60 s.

**Table 1:** Primers and probes used in amplification of Chikungunya RNA as previously described by Laurent et al. 2007 [28] (modifications of primers are indicated in bold).

Name	Sequences (5′→3′)	Sense	Position
CHIKV UBIV F	AAGCT**Y**CGCGTCCTTTACCAAG	Forward	10366–10387
CHIKV UBIV R	CCAAATTGTCC**Y**GGTCTTCCT	Reverse	10574–10554
CHIK PROBE	(FAM)-CCAATGTCTTCAGCCTGGACACCTTT-(Tamra)	Forward	10486–10511

Positive controls, negative controls and no template controls (NTC) were included in each series. Runs were validated only if the NTC and the negative control did not exhibit fluorescence curves that crossed the threshold line, and the positive control gave a fluorescence curve that crossed the threshold line within 39 cycles (Ct≤39). A specimen was considered positive for CHIKV if it gave a positive reaction with a Ct≤39.

### Evaluation of differences in viral quantity between different sampling methods

Dilutions (10^−3^ to 10^−7^) of CHIKV-SP were mixed with blood from a healthy donor. Fifteen µL of this mix was blotted on Whatman 3 MM filter Paper and a 6 mm diameter disk containing the dried spot was cut. Viral RNA was extracted and detected in blood samples mixed with different dilutions of CHIKV-SP before (15 µL) and after being blotted onto filter papers.

### Statistical analysis

The sensitivity and specificity of the assays were evaluated using two-by-two tables. The sensitivity and specificity of the DBFP specimens were determined by comparison with the results obtained with venous blood samples (sera) by the routine diagnostic test (real-time RT-PCR). Data were recorded and analysed statistically by R Analysis with R version 2.7.0 software [29]. Results with a two-sided p value≤0.05 were scored as being significant. A non-parametric test, the Wilcoxon test, was also carried out. Receiver Operating Characteristic (ROC) plot analysis was performed to determine the best threshold value for the CHIKV RNA load by real-time RT-PCR obtained with DBFP compared with the values obtained from sera. As the mean value for the negative reference sample is expected to be smaller than the mean value for the positive reference sample, inverse transformation of the test data was used to prepare data for analysis as previously described [30]. AUC (Area under Curve) values were used to assess the discrimination of the test compared with the reference.

## Results

### Characteristics of patients and specimens

During the first week of February 2010, the health authorities of the health district of Mananjary (Southeast coast of Madagascar) reported an increased incidence of febrile syndrome with arthralgia. Sera from 11 suspected cases were shipped to NRLA at the Institut Pasteur from Madagascar. All specimens tested positive for CHIKV by real-time RT-PCR. From February to October 2010, 3,177 suspected cases were recorded by DULMN and 191 cases were confirmed by laboratory tests.

Overall, 181 samples from patients presenting dengue-like illness were included in our study: 73 (40.3%) were CHIKV confirmed cases and 108 (59.7%) were negative controls; the median age were 18 years and 32 years and sex ratio (M/F) were 1.2 and 0.5, respectively. No dengue virus infection was detected.

### Comparison between sera and DBFP specimens

Among the 181 patients tested, DBFP for 74 (40.9%) and sera for 73 (40.3%) scored positive for CHIKV (Table 2). Results for DBFP and sera were concordant for 170 (93.9%) patients and discordant for 11 (6.1%). The Kappa coefficient was 0.87 (p<0.001; 95% CI:[0.80–0.95]). The sensitivity and the specificity of the test performed with DBFP were 93.1% (68/73; 95% CI:[84.7–97.7]) and 94.4% (102/108; 95% CI:[88.3–97.9]), respectively (Table 2).

**Table 2:** Sensitivity and specificity of test performed on DBFP for the diagnosis of Chikungunya virus infection^a^.

	Result of standard method of virus detection with venous blood samples	
Virus detection in DBFP	Positive	Negative
Positive	68	6
Negative	5	102

a Sensitivity, 93.1% (68/73, (95% CI:[84.7–97.7]); specificity, 94.4% (102/108, (95% CI:[88.3–97.7]); Kappa coefficient, 0.87 (95% CI:[0.80–0.94]).

### Evaluation of differences in viral quantity between different sampling methods

**Figure 1:**
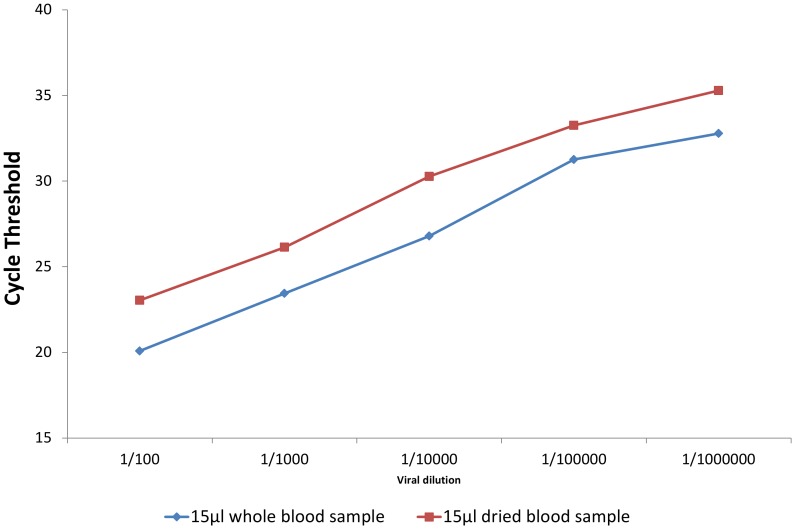
Ct Differences in RNA amplification from 15 µl aliquots of whole blood and DBFP.

The quantities of RNA obtained from filter paper and serum are shown in Figure 1. The difference of the Cycle threshold (Ct) between viral amplification from 15 µl of whole blood and DBFP containing the same viral dilution varied from 2.5 to 3.48. As 3.32 Ct is equivalent to 1 Log of RNA quantity, the loss of viral RNA associated with drying samples on filter paper can be estimated to be between 0.5 Log to 1.05 Log (mean 0.825 Log).

### Determination of Ct cut-off value (Ct-off) for real-time PCR with DBFP samples

**Figure 2:**
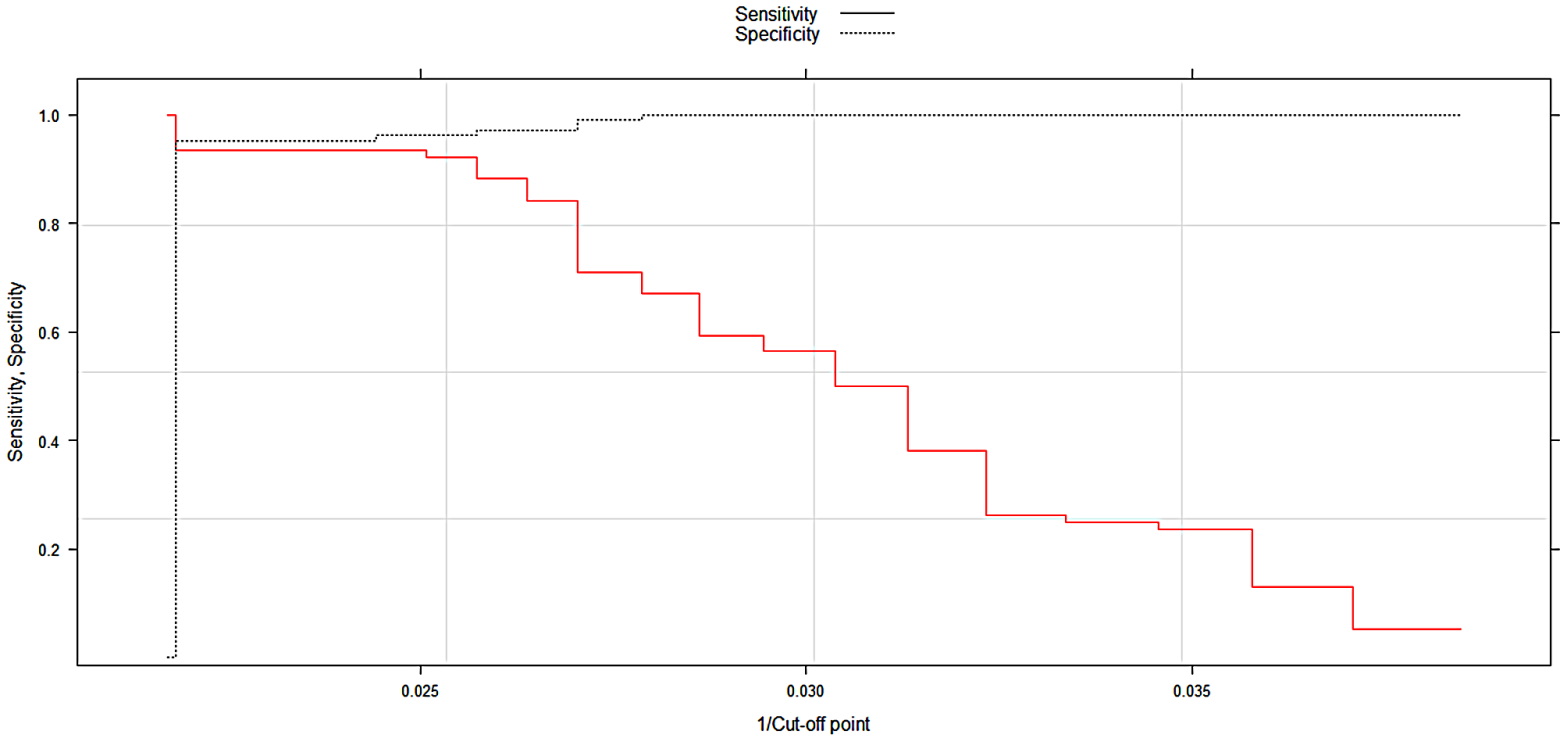
ROC curve to define the threshold of positivity for DBFP.

The value of the Area Under Curve (AUC) was 0.96 (95% CI:[0.93–0.99]. The optimal Ct cut-off for real-time RT-PCR with dried blood spot samples was 40 with an AUC = 0.96; this value correctly classified 95.3% of the data (sensitivity = 93.4% and specificity = 96.2%) (Figure 2).

All 11 discordant cases had Ct values between 37 and 39 cycles. A second real-time RT-PCR was performed in triplicate for 8 of these 11 discordant paired samples and both concordant and discordant results were obtained with the triplicate repeats (data not shown).

## Discussion

The aim of this study was to validate an alternative method for sampling and the diagnosis of CHIKV infection suitable for surveillance in low income countries. Serological methods (IgM detection) have been used to confirm CHIKV infection or circulation [23], but these methods have various limitations: for example, it is generally necessary to obtain paired sera, one during the acute phase and one during the convalescent phase such that seroconversion or an increase in IgM titres can be detected. In most of poor settings countries like Madagascar or in remote regions, it can be difficult to obtain serum during the convalescent phase. The use of molecular techniques has already been described for the diagnosis of infection caused by several viruses, including dengue [21], [22], measles [31], and Rift Valley fever virus [32]. Genotyping of measles virus has also been reported [33].

Our study showed that for diagnosis, outbreak monitoring and virological surveillance of CHIKV infection and circulation, capillary blood samples taken from the finger and spotted onto filter paper is a cost-effective alternative with a good sensitivity and specificity (93.1% and 94.4%, respectively). Together with the detection of Dengue virus from DBFP, that has been found to perform well with a sensitivity and a specificity of 90.7% and 82.9% respectively when compared to the detection from sera [21], [22]. We implement our sentinel surveillance for the detection of both CHIKV and Dengue Virus using DBFP as specimen collection system. Despite its good sensitivity, we have observed some discordant results between DBFP and sera. One explanation could be an intrinsic low viral load in the samples and the limit of detection of the real-time RT-PCR. A similar observation has been reported in other studies [22]. Viral RNA in DBFP may decay, if stored for long periods, and this is a possible limitation, as the virus may become undetectable. However, some studies have shown that viral RNA in filter paper was stable for several weeks at room temperature (25°C) [26]. In our laboratory, we were able to detect CHIKV in DBFP after 6 months of storage at 25°C (data not shown). Similarly, Gauffin F. et al. found no significant decay of RNA in dried blood spots stored for up to 20 years [34].

Another possible limitation of our study is that the viral load may differ between capillary and venous blood. However, this does not appear to be a problem because we restricted blood collection to the first 5 days after the onset of fever. Nevertheless, both the decay of CHIKV RNA upon storage and viral loads in capillary and venous blood should be further studied.

The use of DBFP instead of serum eliminates the need for a cold chain or for a nitrogen tank for transportation. For our current arbovirus surveillance system, the overall cost for sampling and shipping of specimens using a 10 L nitrogen tank by road once a week from/to one sentinel site is around 300 US$ per week whereas using DBFP this cost has decreased to less than 10 US$ per week. For centres only accessible by air, costs of shipment using nitrogen tank or isothermal boxes is not sustainable for a country like Madagascar. This method has other advantages. In particular, capillary blood collection is easier in young children. Self-collection is also possible during outbreaks when health workers may be overloaded with work. Self-collection of DBFP samples has already been used successfully for serosurveys during the 2006 Chikungunya outbreak in La Réunion [23]. Currently, this method of collection is used by the Ministry of Health from the Union of the Comoros to collect and ship specimen to our NRLA for dengue-like syndromes investigations.

Despite these various advantages of the use of DBFP, it is important to note that this method is probably not suitable for subsequent analyses involving growing the virus. Nevertheless, it has been shown that recovery of some flaviviruses (Dengue, West Nile and Yellow fever) and an alphavirus (Venezuelan equine encephalitis) was possible from DBFP stored for up to 90 days [12], [26]. More work is needed to evaluate the viability of CHIKV in dried blood.

In conclusion, we demonstrate that DBFP is a cost-effective method for surveillance and for the monitoring of viral outbreaks in low income countries, and especially in large countries where the access to laboratory facilities is limited. This method can facilitate the extension of surveillance system networks, and may be useful to public health authorities for rapid identification of Chikungunya outbreaks and, by extension, those of other arboviruses (e.g. Dengue fever, Rift Valley Fever, West Nile).

## Supporting Information

Checklist S1 STARD checklist.
(DOC)

Flowchart S1 STARD flow chart detailing individuals recruited for this study, and the order of RT-PCR execution.
(DOC)
