# Supplementary material for: Dried-Blood Spots: A Cost-Effective Field Method for the Detection of Chikungunya Virus Circulation in Remote Areas
Source: PLoS Negl Trop Dis. 2013 Jul 25;7(7):e2339. doi: 10.1371/journal.pntd.0002339 (PMC3723542; doi:10.1371/journal.pntd.0002339)
Supplement: Flowchart S1 — STARD flow chart detailing individuals recruited for this study, and the order of RT-PCR execution. (DOC) [file pntd.0002339.s002.doc]

**Figure S1:** Study design flowchart

Suspected dengue-like syndrome

(**n=3177**)

**Excluded patients (n=2996)**

- Sample not available (n=2826)
- DBFP not available (n= 170)

- Confirmed CHIKV (n=118)

- Negative (n=52)

Included patients

(**n=181**)

Positive = cases

(**n=73**)

Negative = control

(**n=108**)

Positive

(**n=68**)

Negative

(**n=5**)

Real-time RT-PCR – sera

Negative

(**n=102**)

Positive

(**n=6**)

Real-time RT-PCR - DBFP
